# Association of the Lipoprotein Receptor *SCARB1* Common Missense Variant rs4238001 with Incident Coronary Heart Disease

**DOI:** 10.1371/journal.pone.0125497

**Published:** 2015-05-20

**Authors:** Ani Manichaikul, Xin-Qun Wang, Solomon K. Musani, David M. Herrington, Wendy S. Post, James G. Wilson, Stephen S. Rich, Annabelle Rodriguez

**Affiliations:** 1 Center for Public Health Genomics, University of Virginia, Charlottesville, Virginia, United States of America; 2 Department of Public Health Sciences, Biostatistics Section, University of Virginia, Charlottesville, Virginia, United States of America; 3 Department of Medicine, University of Mississippi Medical Center, Jackson, Mississippi, United States of America; 4 Department of Internal Medicine, Wake Forest University School of Medicine, Winston-Salem, North Carolina, United States of America; 5 Department of Medicine, Johns Hopkins University School of Medicine, Department of Epidemiology, Johns Hopkins Bloomberg School of Public Health, Baltimore, Maryland, United States of America; 6 Department of Physiology and Biophysics, University of Mississippi Medical Center, Jackson, Mississippi, United States of America; 7 Department of Cell Biology, University of Connecticut Health Center, Farmington, Connecticut, United States of America; Universite de Montreal, CANADA

## Abstract

**Background:**

Previous studies in mice and humans have implicated the lipoprotein receptor *SCARB1* in association with atherosclerosis and lipid levels. In the current study, we sought to examine association of *SCARB1* missense single nucleotide polymorphism (SNP) rs4238001 with incident coronary heart disease (CHD).

**Methods and Results:**

Genotypes for rs4238001 were imputed for 2,319 White, 1,570 African American, and 1,292 Hispanic-American MESA participants using the 1,000 Genomes reference set. Cox proportional hazards models were used to determine association of rs4238001 with incident CHD, with adjustments for age, sex, study site, principal components of ancestry, body mass index, diabetes status, serum creatinine, lipid levels, hypertension status, education and smoking exposure. Meta-analysis across race/ethnic groups within MESA showed statistically significant association of the T allele with higher risk of CHD under a consistent and formally adjudicated definition of CHD events in this contemporary cohort study (hazard ratio [HR]=1.49, 95% CI [1.04, 2.14], *P* = 0.028). Analyses combining MESA with additional population-based cohorts expanded our samples in Whites (total n = 11,957 with 871 CHD events) and African Americans (total n = 5,962 with 355 CHD events) and confirmed an increased risk of CHD overall (HR of 1.19 with 95% CI [1.04, 1.37], *P* = 0.013), in African Americans (HR of 1.49 with 95% CI [1.07, 2.06], *P* = 0.019), in males (HR of 1.29 with 95% CI [1.08, 1.54], *P* = 4.91x10^-3^) and in White males (HR of 1.24 with 95% CI [1.03, 1.51], *P* = 0.026).

**Conclusion:**

*SCARB1* missense rs4238001 is statistically significantly associated with incident CHD across a large population of multiple race/ethnic groups.

## Introduction

Deficiency of the scavenger receptor class B type I (SR-BI) in mice is significantly associated with abnormal lipoprotein composition (especially LDL-cholesterol [LDL-C] and HDL-cholesterol [HDL-C]) and accelerated atherosclerosis in the background of *ApoE-/-* or *ldlr*-/-, among other phenotypic changes [1–5]. Rigotti *et al*. [6] showed that plasma total cholesterol levels were significantly elevated in SR-BI knockout (KO) mice as compared with wild-type mice. Using fast protein liquid chromatography, these investigators observed that the HDL profiles were very different, with a heterogeneous peak and enrichment of apoE. Similar differences in the IDL/LDL profiles were also observed in the SR-BI KO mice. Braun *et al*. [7] subsequently reported the association of SR-BI deficiency in mice with early onset atherosclerotic disease.

Many of these phenotypic changes have now been identified in humans who are carriers of certain *SCARB1* single nucleotide polymorphisms (SNPs). In one of the earlier observations, Acton *et al*. [8] reported that the missense rs4238001 variant, which is a nonsynonymous coding SNP in exon 1 that encodes an amino acid change at the second position from glycine to serine (p.Gly2Ser), was significantly associated with higher levels of HDL-C and lower levels of LDL-C in males, with no association observed in females. More recently, West *et al*. [9] showed that SR-BI protein expression was an independent predictor of HDL-C levels in subjects with hyperalphalipoproteinemia. Furthermore, these investigators showed that body mass index (BMI) and rs4238001 were independent predictors of SR-BI protein levels. Of note, subjects who were carriers of the risk T allele for rs4238001 had significantly lower SR-BI protein levels as compared with carriers of the referent allele. Moreover, *in vitro* approaches showed that the rs4238001 variant was significantly associated with increased degradation of SR-BI protein and reduced function as measured by decreased specific cholesteryl ester uptake from HDL [9].

Our previous genetic analyses of *SCARB1* variants in MESA participants [10,11] did not examine the common functional polymorphism rs4238001, as it was neither genotyped in the cohort, nor available in our imputation of SNPs from the HapMap I+II reference panel [12]. Subsequently, the 1,000 Genomes project [13] characterized an even broader set of SNPs than previously available in the HapMap, making it possible for us to carry out, for the first time, this hypothesis driven association analysis of the primary candidate *SCARB1* SNP rs4238001 with incident CHD in MESA participants. We focus the current investigation on the single SNP rs4238001, as it was the only common *SCARB1* missense variant reported in the Exome Variant Server (http://evs.gs.washington.edu/EVS/) with minor allele frequency (MAF) > 5%. In addition to primary genetic association analysis, we also examined the role of traditional risk factors, such as lipids (HDL-C and LDL-C), and non-traditional risk factors, such as lipoprotein subfractions and inflammatory biomarkers, in the causal pathway of rs4238001 with CHD outcomes.

While performing analyses within MESA allowed for uniformity in definitions of CHD and extended regression modeling using the rich set of additional risk factors available within the cohort, we recognized the importance of examining the association of rs4238001 with CHD in a larger set of population-based samples. Therefore, we expanded our main association analysis to incorporate participants from three additional cohorts. Combining these additional cohorts with participants from MESA, our investigation represented a total of n = 11,957 Whites (with n = 871 CHD cases) and n = 5,962 African Americans (with n = 355 CHD cases), and n = 1,255 Hispanics (with n = 39 CHD cases) in the fully adjusted regression analyses. The larger sample size was particularly important in providing improved power to examine the effects of rs4238001 in race/ethnic- and sex-specific stratified analyses. We emphasize, however, that the primary aim of the current study is to examine the evidence of association between rs4238001 and CHD overall, with race/ethnic- and sex-specific analyses constituting secondary analyses for the current effort. Our study represents a careful and detailed characterization of clinically relevant cardiovascular endpoints (e.g. CHD) for a missense SNP with previously demonstrated functional effects on SR-BI degradation and cholesteryl ester uptake [9].

## Methods

### Ethics statement

All MESA participants gave written informed consent, including consent to participate in genetic studies. The MESA study was approved by the Institutional Review Boards of the National Heart Lung and Blood Institute and all participating institutions, including Wake Forest University, Columbia University, Johns Hopkins University, the University of Minnesota, Northwestern University, the University of California—Los Angeles, the Cedars-Sinai Medical Center and the University of Virginia. All methodology was compliant with the principles set forth in the Declaration of Helsinki.

### Study Design

The Multi-Ethnic Study of Atherosclerosis (MESA) is a longitudinal study of subclinical atherosclerosis (SCA) and risk factors that predict progression to clinically overt cardiovascular disease (CVD) or progression of the subclinical disease [14]. The first clinic visits occurred in 2000–2002 in 6,814 participants recruited from six field centers across the United States, and all participants were free of CVD at the baseline exam. Approximately 38% of the recruited participants were White, 28% African American, 22% Hispanic, and 12% Asian, predominantly of Chinese descent. MESA was approved by the IRB at all participating sites, and all participants gave informed consent.

### Phenotyping of MESA participants

MESA participants had detailed medical histories (including medication and smoking history) and underwent examinations for anthropometry, blood pressure and vascular imaging. Fasting blood samples were taken for DNA, lipids and inflammatory biomarkers. Cardiovascular events were adjudicated by a MESA committee, in a process that has already been published [15].

### Genotyping and imputation

Participants recruited for the original MESA cohort (n = 6,814) were genotyped in 2009 using the Affymetrix Human SNP array 6.0. Genotype quality control for these data has been described previously [11]. Because the *SCARB1* SNP rs4238001 was not genotyped in MESA, we imputed the genotypes for MESA participants using the available genome-wide genotypes. IMPUTE version 2.2.2 [16] was used to perform imputation for the MESA SHARe participants using the 1,000 Genomes cosmopolitan Phase 1 v3 reference panel [13] and the SNP was imputed with good quality (observed-expected variance ratio > 0.8) in MESA Whites, African Americans and Hispanics. MESA Chinese were not included in the current investigation because the allele frequency in this group did not pass our inclusion threshold of MAF > 0.05. We further validated the quality of imputation by comparison with genotypes obtained by direct exome sequencing through the NHLBI Exome Sequencing Project for a subset of 399 MESA participants (251 White and 148 African American) [17]. Among these participants, we observed 97.1% and 99.3% concordance in Whites and African Americans, respectively, comparing best guess genotypes obtained by imputation with those obtained directly by exome sequencing.

### Genetic association analysis in MESA

We began with stratified analyses within each race/ethnic group in MESA. We performed Cox proportional hazards analysis of incident CHD with respect to rs4238001 genotype under an additive 1 df genetic dosage model for the risk allele T. We began with a basic regression model (Model 1) that included adjustment for age, sex, study site and principal components of ancestry, an extended model that added adjustment for major coronary risk factors (Model 2) including body mass index (BMI), diabetes status, serum creatinine, LDL-C, HDL-C, hypertension status, education, and smoking exposure (ever smoke and current smoke), a model that extended Model 2 with adjustment for statin use (Model 3), and a model that added to Model 2 adjustment for HDL and LDL particle number and size from NMR spectroscopy (Model 4). These regression analyses were further stratified by sex for each race/ethnic group.

Following race/ethnic stratified analyses, we performed fixed effect meta-analysis to combine results across all three race/ethnic groups in METAL [18]. We designated the pooled analyses (of males and females) under Models 1 and 2 as our primary analyses. We report all *P*-values <0.05 from regression analyses for primary analyses. Additional regression Models 3 and 4, and sex-stratified analyses are designated as secondary analyses. We report results from secondary analyses in order to interpret the results from primary analyses.

### Validation cohorts

We sought validation of the observed associations for the rs4238001 SNP through expanded analysis incorporating independent cohorts spanning both White and African American populations, including the Atherosclerosis Risk in Communities Study (ARIC), the Framingham Heart Study (FHS) and the Jackson Heart Study (JHS) for whom GWAS genotyping were available to allow imputation of the rs4238001 SNP. Additional details are described in **S1 Supporting Information**.

## Results

### Characteristics of the MESA study samples

The MESA participants included in our genetic association analyses included 2,319 Whites, 1,570 African Americans, and 1,292 Hispanics, roughly evenly distributed between males and females. The median age of participants in each ethnic group ranged from 61–63 years, with an interquartile range (IQR) of 53–71 years of age. Body mass index values were highest in African Americans (median [IQR] of 29.4 [26.1, 33.6] kg/m^2^) and lowest among Whites (27.0 [24.2, 30.3] kg/m^2^). Triglyceride levels were notably lower in African Americans (90 [66, 123] mg/dl) compared with Hispanics (136 [96, 192] mg/dl), while HDL-C, LDL-C, and serum creatinine measures were qualitatively similar across ethnic groups. At baseline, Whites demonstrated lower prevalence of diabetes (5.6%) than both African Americans (17.4%) and Hispanics (17.7%). Hypertension was also less frequent in Whites (38.8%) compared to African Americans (59.4%) and Hispanics (42.6%). Rates of current smoking were <20% in each of the three ethnic groups. Lipid medication use was also <20% in each of the three ethnic groups included in the current investigation (**Table 1**). Clinical events were assessed after a median 7.6 years of follow-up by a MESA adjudication committee that applied a uniform definition of events to all participants that included incident MI, definite angina, probable angina (if followed by coronary artery bypass grafting or percutaneous coronary intervention), resuscitated cardiac arrest, or coronary heart disease death. At this point in the study, the cumulative incidence of probable or confirmed CHD events (CHD-All) was 6.1%, 4.6% and 4.6% in Whites, African Americans and Hispanics, respectively, while the cumulative incidence of confirmed CHD events (CHD-Hard) was 3.3%, 2.9% and 3.1%, respectively (**Table 1**). Rates of CHD events were consistently higher in males (4.7%, 4.1% and 4.6%) than females (2.1%, 1.9%, 1.7%) in Whites, African Americans and Hispanics.

**Table 1:** Characteristics of MESA participants across three ethnic groups.

	White	African American	Hispanic	*P*-value†
**Participant characteristics** *	** **		** **	** **
No. subjects	2319	1570	1292	—-
Women	1208 (52.1)	841 (53.6)	655 (50.7)	0.308
Age, years	63 [54, 71]	63 [53, 70]	61 [53, 69]	**<0.001**
BMI, kg/m^2^	27.0 [24.2, 30.3]	29.4 [26.1, 33.6]	28.6 [26.0, 31.8]	**<0.001**
Education: completed high school	2199 (95.1)	1369 (87.9)	684 (52.9)	**<0.001**
Education: completed technical degree, associate degree, bachelor's degree or higher	1410 (61.0)	749 (48.1)	273 (21.1)	**<0.001**
				
Triglycerides, mg/dL	114 [77, 164]	90 [66, 123]	136 [96, 192]	**<0.001**
HDL-C, mg/dL	50 [41, 61]	50 [41, 60]	45 [39, 54]	**<0.001**
LDL-C, mg/dL	115 [95, 136]	115 [95, 136]	119 [98, 139]	**0.003**
Serum creatinine, mg/dL	0.90 [0.80, 1.10]	1.00 [0.90, 1.10]	0.90 [0.80, 1.00]	**<0.001**
				
Diabetes (yes/no)	130 (5.6)	272 (17.4)	228 (17.7)	**<0.001**
Hypertension (yes/no)	899 (38.8)	932 (59.4)	550 (42.6)	**<0.001**
				
Ever smoke (yes/no)	1287 (55.7)	851 (54.7)	593 (45.9)	**<0.001**
Current smoke (yes/no)	263 (11.3)	290 (18.5)	175 (13.5)	**<0.001**
				
Lipid medication (yes/no)	421 (18.2)	246 (15.7)	177 (13.7)	**0.002**
				
**Clinical events**				
Coronary heart disease—All	142 (6.1)	73 (4.6)	60 (4.6)	0.063
Coronary heart disease—Hard	77 (3.3)	46 (2.9)	40 (3.1)	0.785
Myocardial infarction	63 (2.7)	24 (1.5)	34 (2.6)	**0.031**
Follow-up time (years)	7.6 [7.4, 7.8]	7.5 [7.0, 7.7]	7.6 [7.1, 7.8]	**<0.001**

Data are presented as N (%) for binary measures or median [IQR] for continuous measure.

*Summary statistics are reported for the subset of individuals with data available for at least one of the clinical events.

†P-values are presented for statistical significance of the difference in values across race/ethnic groups according to a likelihood ratio test with 2 degrees of freedom.

### *SCARB1* rs4238001 SNP and CHD in MESA

Since we had previously reported that rs4238001 was an independent predictor of SR-BI protein and that this variant was significantly associated with accelerated SR-BI protein degradation and reduced cholesteryl ester uptake, the next logical step was to assess its association with hard clinical CVD endpoints. Thus, the current investigation begins with a known testable hypothesis rather than a discovery effort as often seen in GWAS and related genetic screening approaches. Therefore, we did not employ any correction for multiple comparisons, and used the nominal threshold of *P* = 0.05 for statistical significance. The primary hypothesis for the current investigation is that rs4238001 genotype is associated with CHD in the general population. Stratified analyses by sex and race/ethnicity are secondary to this primary hypothesis.

**Fig 1:**
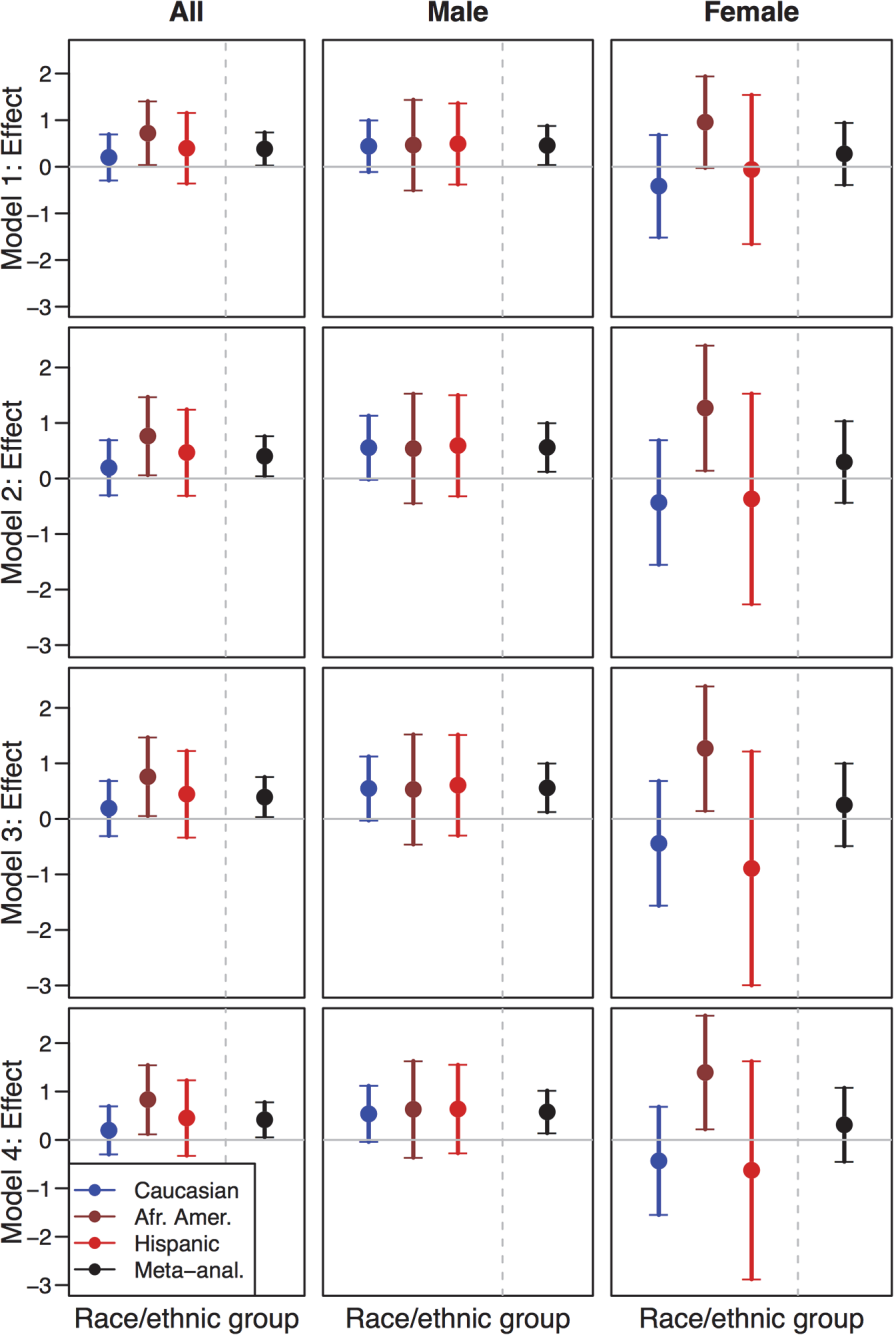
Effect estimates (log hazard ratio of CHD for rs4238001 effect allele T) and corresponding 95% confidence intervals shown for Model 1 (basic), Model 2 (extended), Model 3 (Model 2 + lipid medication) and Model 4 (NMR lipids). Analyses were conducted stratified by race/ethnic group and combined by meta- analysis, for all participants as well as stratified by sex (males or female).

In meta-analysis, we observed a statistically significant increase in the risk of CHD-Hard for the risk T allele (**S1 Table**, hazard ratio [HR] of 1.49, 95% CI [1.04, 2.14], *P* = 0.028) under a regression model that includes adjustment for major coronary risk factors (Model 2). In stratified analyses by race/ethnic group, we observed statistically significant association of the risk T allele in African Americans (**S2 Table**, Model 2 HR of 2.15 (95% CI [1.06, 4.35], *P* = 0.033). We did not observe significant association of CHD-Hard with the risk T allele in Whites, nor in Hispanics (**S2 Table**). We reiterate, however, that the risk T allele showed a significant association with increased risk of CHD events in meta-analysis across race/ethnic groups and the increase in risk with the T allele was observed consistently across all three race/ethnic groups (heterogeneity *P*-value = 0.42). Sensitivity analyses showed our finding were robust to multiple models of adjustment (**S1 Table and S2 Table, Fig 1**).

We examined evidence of association for rs4238001 with CHD in meta-analysis that combined sex-specific results across the three race/ethnic groups. In males, we observed statistically significant association with CHD-Hard (**S1 Table**, Model 2 HR = 1.76, 95% CI [1.14, 2.71], *P* = 0.011). We observed hazard ratios of the same direction and similar magnitude in both males and females (heterogeneity *P*-value = 0.54). We did not observe any statistically significant association at the nominal level (α = 0.05) in race/ethnic-specific analysis of males. In African American females, we observed statistically significant evidence of association with CHD-Hard (**S2 Table**, Model 2 HR = 3.59, 95% CI [1.17, 11.07], *P* = 0.027). This result corresponds to an odds ratio of 3.98 (95% CI [1.18, 13.47], *P* = 0.026) for increased risk of CHD-Hard among African American females. We emphasize, however, that sex-stratified results within race/ethnic groups represent relatively fewer CHD cases (**S2 Table**). In addition, consideration of multiple regression models and stratified analyses goes beyond the strict evaluation of our primary hypothesis for association of rs4238001 genotype with CHD overall under a controlled type I error rate of α = 0.05. Accordingly, the statistically significant result in African American females should be interpreted with caution.

### Lipid subfractions and biomarkers

**Fig 2:**
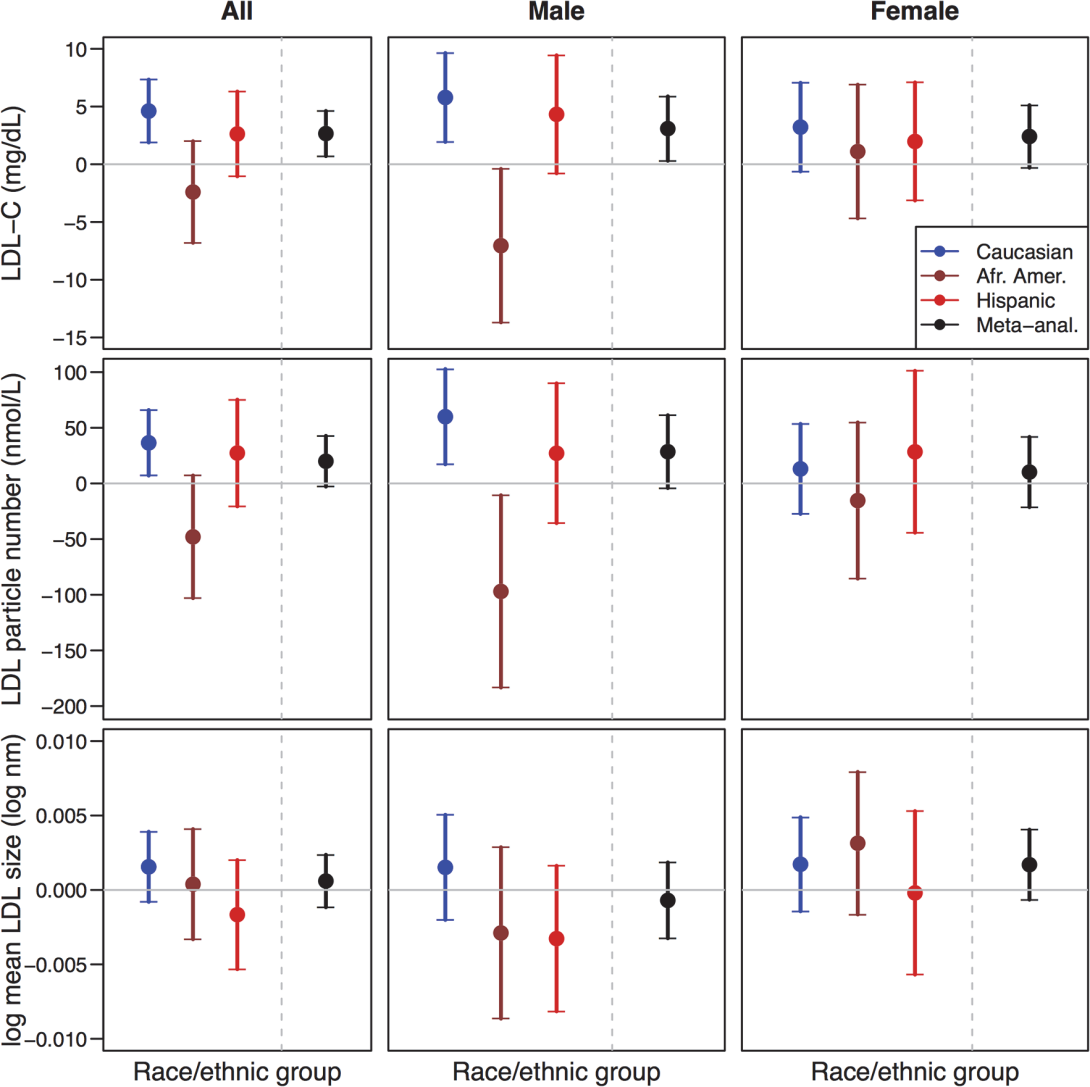
Summary of estimated genetic additive effects of rs4238001 allele T on LDL-C (mg/dL), LDL particle number (nmol/L) and LDL particle size (log nm) under a basic regression model (Model 1). Analyses were conducted stratified by race/ethnic group and combined by meta-analysis, for all participants as well as stratified by sex (males or female).

The association of rs4238001 with CHD-Hard was significant even after multiple adjustments with traditional CVD risk factors including lipids. Therefore, we examined the association of rs4238001 with lipids and subfractions in the three MESA race/ethnic groups. As shown in **Fig 2**, combined meta-analysis across race/ethnic groups showed LDL-C levels were higher in carriers of the risk T allele. In race/ethnic specific analysis, LDL-C levels were higher in White males, lower in African American males, and not statistically significantly different in Hispanic male carriers of the risk allele. We did not observe statistically significant association of rs4238001 with LDL-C levels in females. LDL particle numbers were nominally significantly higher in White males, lower in African American males and not different in Hispanic male carriers.

**Fig 3:**
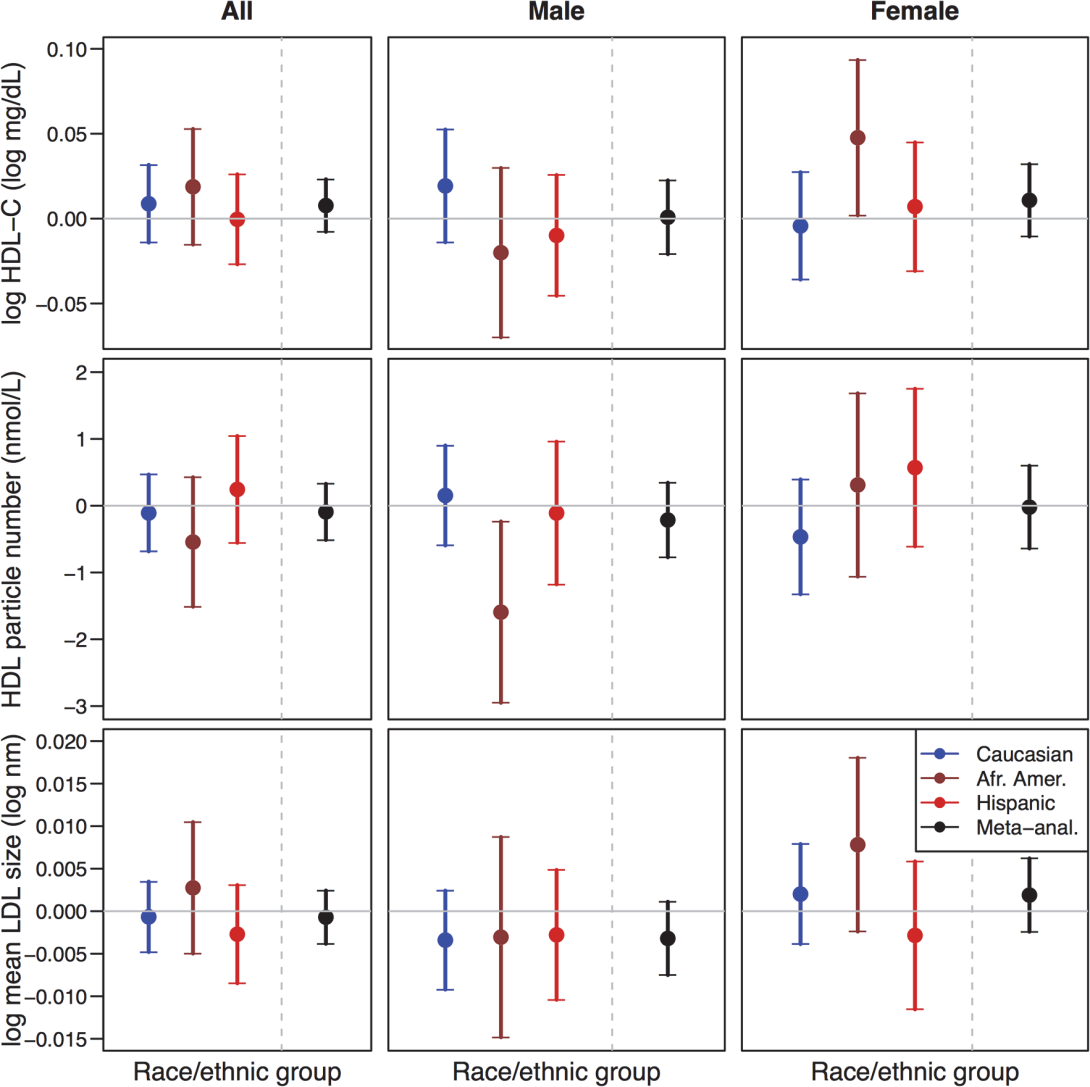
Summary of genetic additive effects of rs4238001 allele T on HDL-C (log mg/dL), HDL particle number (nmol/L) and HDL particle size (log nm) under a basic model (Model 1). Analyses were conducted stratified by race/ethnic group and combined by meta-analysis, for all participants as well as stratified by sex (males or female).

Overall, we did not observe strong differences in mean HDL-C levels with respect to rs4238001 genotypes (**S3 Table**). Using a formal regression model to examine association of HDL parameters with rs4238001 genotypes, we did not observe statistically significant association with HDL-C levels in male carriers of the risk allele (**Fig 3**). However, HDL-C levels were significantly higher in African American female carriers compared to homozygotes for the reference allele. For HDL particle number, we observed statistically significantly lower levels in African American male carriers of the risk allele. There were no significant differences with HDL particle size in either sex.

We next examined the association of the risk allele with biomarkers of inflammation and thrombosis. The results showed that the risk T allele did not significantly affect levels of interleukin-6, eSelectin1, sICAM1, PAI-1, hsCRP or homocysteine, regardless of race or sex stratification (**S4 Table**).

### Expanded analysis with additional Whites and African Americans

Building on the results from MESA, we proceeded to examine associations with CHD events in independent cohorts of Whites from the Atherosclerosis Risk in Communities (ARIC) Study and the Framingham Heart Study (FHS) and African Americans from ARIC and the Jackson Heart Study (JHS) (**S5 Table**). To obtain a clean set of events in the validation cohorts, we applied a strict definition of CHD events to include MI or CHD death.

Combining MESA with additional population-based cohorts expanded our samples in Whites (total n = 11,957 with 871 CHD events across three cohorts [MESA, ARIC and FHS]) and African Americans (total n = 5,962 with 355 CHD events across three cohorts [MESA, ARIC and JHS]). Incorporating MESA Hispanics, the current investigation included a total of n = 19,174 participants with 1265 CHD cases. In meta-analysis across race/ethnic groups, we confirmed an increased risk of CHD overall (**Table 2**, HR of 1.19 with 95% CI [1.04, 1.37], *P* = 0.013) and in males (HR of 1.29 with 95% CI [1.08, 1.54], *P* = 4.91x10^-3^). We further observed statistically significant evidence of association in race/ethnic specific analysis of African Americans (HR of 1.49 with 95% CI [1.07, 2.06], *P* = 0.019) and in White males (HR of 1.24 with 95% CI [1.03, 1.51], *P* = 0.026). Notably, the observed direction of effect for the risk T allele was consistent across all race/ethnic groups and sex-strata examined in our expanded analyses (**Table 2**).

**Table 2:** Association of rs4238001 with CHD events for MESA and combined with participants from additional cohorts in Whites and African Americans.

		rs4238001	MESA			Additional cohorts*			Combined (MESA + Additional cohorts)		
	Group	MAF†	N (events)	Hazard Ratio (95% CI)	P-value	N (events)	Hazard Ratio (95% CI)	P-value	N (events)	Hazard Ratio (95% CI)	P-value
All	White	0.104	2275 (77)	1.214 (0.739, 1.993)	0.442	9682 (794)	1.111 (0.944, 1.307)	0.205	11957 (871)	1.126 (0.97, 1.307)	0.119
	African American	0.055	1533 (46)	2.151 (1.064, 4.348)	0.033	4429 (309)	1.339 (0.923, 1.943)	0.125	**5962 (355)**	**1.485 (1.068, 2.063)**	**0.019**
	Hispanic	0.098	1255 (39)	1.639 (0.756, 3.554)	0.235	-	-	-	-	-	-
	Meta-analysis		5063 (162)	1.495 (1.044, 2.14)	0.028	14111 (1103)	1.145 (0.986, 1.328)	0.076	**19174 (1265)**	**1.191 (1.039, 1.366)**	**0.013**
Male	White	0.103	1088 (52)	1.744 (0.982, 3.096)	0.058	4314 (502)	1.192 (0.973, 1.462)	0.092	**5402 (554)**	**1.244 (1.026, 1.507)**	**0.026**
	African American	0.053	715 (30)	1.714 (0.64, 4.595)	0.284	1697 (145)	1.432 (0.768, 2.671)	0.259	2412 (175)	1.508 (0.89, 2.555)	0.127
	Hispanic	0.103	618 (29)	1.809 (0.73, 4.484)	0.200	-	-	-	-	-	-
	Meta-analysis		2421 (111)	1.754 (1.135, 2.71)	0.011	6011 (647)	1.214 (1.000, 1.474)	0.051	**8432 (758)**	**1.289 (1.079, 1.541)**	**4.91x10** ^**-3**^
Female	White	0.105	1187 (25)	0.649 (0.212, 1.988)	0.449	5368 (292)	1.033 (0.783, 1.361)	0.819	6555 (317)	1.006 (0.769, 1.316)	0.966
	African American	0.056	818 (16)	3.593 (1.166, 11.068)	0.027	2732 (164)	1.252 (0.785, 1.997)	0.343	3550 (180)	1.462 (0.95, 2.251)	0.084
	Hispanic	0.094	637 (10)	0.693 (0.104, 4.61)	0.704	-	-	-	-	-	-
	Meta-analysis		2642 (51)	1.347 (0.649, 2.798)	0.424	8100 (456)	1.087 (0.857, 1.377)	0.496	10742 (507)	1.108 (0.885, 1.389)	0.370

*Additional cohorts include ARIC and FHS for Whites, and ARIC and JHS for African Americans.

†The frequency of rs4238001 risk T allele is reported for all participants from the contributing cohorts for the stated race/ethnic group.

Regression analysis results are based on Cox proportional hazards models of CHD for rs4238001 (effect allele T) including the covariates for Model 2: age, sex, principal components of ancestry, study site (cohort specific), body mass index (BMI), diabetes status (yes/no), serum creatinine, LDL-C, HDL-C, hypertension status (yes/no), education, smoking exposure (ever smoke [yes/no] and current smoke [yes/no]). Hazard ratios are presented based on an additive 1 df dosage model for the number of copies of the risk T allele, with common allele homozygotes CC as the reference group.

Combining MESA with additional population-based cohorts expanded our samples in Whites (total n = 11,957 with 871 CHD events across three cohorts [MESA, ARIC and FHS]) and African Americans (total n = 5,962 with 355 CHD events across three cohorts [MESA, ARIC and JHS]). Incorporating MESA Hispanics, the current investigation included a total of n = 19,174 participants with 1265 CHD cases. In meta-analysis across race/ethnic groups, we confirmed an increased risk of CHD overall (**Table 2**, HR of 1.19 with 95% CI [1.04, 1.37], *P* = 0.013) and in males (HR of 1.29 with 95% CI [1.08, 1.54], *P* = 4.91x10^-3^). We further observed statistically significant evidence of association in race/ethnic specific analysis of African Americans (HR of 1.49 with 95% CI [1.07, 2.06], *P* = 0.019) and in White males (HR of 1.24 with 95% CI [1.03, 1.51], *P* = 0.026). Notably, the observed direction of effect for the risk T allele was consistent across all race/ethnic groups and sex-strata examined in our expanded analyses (**Table 2**).

## Discussion

We completed a genetic association study for the common functional polymorphism rs4238001 with CHD and MI events in MESA. In analysis that combined data from Whites, African Americans and Hispanics, we identified significant evidence for the risk T allele as a factor for CHD-Hard events in MESA participants. When combining evidence across all race/ethnic groups from MESA, we observed significant evidence of the rs4238001 SNP as a risk factor for CHD-Hard in males, but not in all female groups. The evidence of association was strongest in African Americans, with consistent direction of risk effect seen in the other ethnic groups.

As the current investigation was hypothesis driven in testing the effect of a known missense mutation in a well-established gene of functional significance, we did not carry out formal correction for multiple comparisons. However, we recognize that we have carried out multiple stratified analyses by sex and race/ethnicity. We emphasize that these stratified analyses are secondary compared to the primary question of whether or not rs4238001 associates with CHD overall. Differences observed across strata in the current study may be taken as hypothesis generating, and will require future hypothesis-driven research for follow-up. Another potential source of multiple testing lies in the multiple regression models under consideration. We emphasize, however, that we have employed only a single model (Model 2) for testing our primary hypothesis. Additional regression models presented in this work are shown for the purpose of sensitivity analysis to examine results with adjustment for additional factors of interest. Notably, we did not observe strong qualitative changes in our results with the different covariate adjustments considered.

In analyses stratified by sex in MESA, the increased risk of CHD in male participants is consistent with well-established observations of higher CHD rates in males [19–20]. In women, meta-analysis did not show a significant association of rs4238001 with CHD. Interestingly, the association of rs4238001 with LDL-C was similarly stronger in males than in females, suggesting the underlying effects of the rs4238001 variant exhibit similar patterns of sex-specificity for both LDL-C and CHD. While the association of rs4238001 with CHD remained significant across multiple regression models with adjustment for traditional risk factors (like HDL-C and LDL-C) and non-traditional risk factors such as lipoprotein particle number/size and education, we do not rule out the possibility of a role for lipids in the effect of rs4238001 on CHD. Indeed, adjustment for baseline LDL-C cannot reflect the full range of effects that LDL-C may have on an individual’s cardiovascular risk throughout the course of a lifetime.

Follow-up including an expanded set of Whites from ARIC and FHS and African Americans from ARIC and JHS provided additional support for the association of rs4238001 with CHD overall, in African Americans, as well as in sex-stratified analyses of males and White males, in particular. The statistical significance within these subgroups reflects, in part, the greater power we had to observe effects in groups with greater sample sizes, higher rates of CHD, and/or higher frequency of the rs4238001 risk allele (**S6 Table**).

The estimated effects observed in the cohorts added at the stage of expanded analyses were generally attenuated compared to those seen in MESA (**Table 2**). The observed differences in effects may reflect in part the Winner’s curse, a phenomenon in which larger effect sizes are seen in discovery cohorts compared to those found in subsequent validation efforts [21]. In addition, we emphasize that MESA has some important differences compared to the other cohorts, including being the only contemporary cohort and having its own internally consistent definitions of hard CHD events that could not be carried over to the expanded analyses. Recognizing that the minor allele frequency for the SNP ranges from 5–10% across race/ethnic groups, and that event counts for carriers of the T allele are small for individual cohorts (**S7 Table**), we caution against systematic interpretation of stratified and cohort-specific results. We further emphasize that the rs4238001 allele T conferred increased risk of CHD in all subgroups examined for our current analyses, and failure to achieve statistical significance within a particular subgroup does not imply a lack of association within that group.

In analyses stratified by race/ethnic group in MESA, the association of rs4238001 with incident CHD was significant in African Americans across the multiple regression models. The effect estimate was positive in all models, which suggests the increased risk for incident CHD in this race/ethnic group was also independent of traditional risk factors. Hurley *et al*. [22] demonstrated that some traditional risk factors, such as HDL, are not strong predictors of CHD in African Americans. Further study of rs4238001 in relation to CHD outcomes may offer the opportunity to identify novel risk factors of particular relevance to African Americans. This area of research may serve to address the higher rate of cardiovascular mortality in minority populations, especially for premature cardiovascular death (<65 years of age) in African Americans [23].

To date there have been few studies that examined the association of *SCARB1* SNPs with prevalent or incident CVD. We recently reported the association of the intronic *SCARB1* SNP rs10846744 with subclinical atherosclerosis and incident CVD [11], and a nearby SNP was also reported among those reaching the FDR threshold of 5% in a recent large-scale GWAS of CHD from the CARDIOGRAMplusC4D consortium [24]. The SNP rs10846744 lies more than 35 kb downstream from rs4238001, and the two *SCARB1* SNPs exhibit very little linkage disequilibrium (R-squared of 0.002, 0.030 and 0.002 for 1,000 Genomes Phase 1 v3 EUR, AFR and AMR samples, respectively). Recently, Rejeb *et al*. [25] reported association of three *SCARB1* SNPs, including rs4238001, as significantly associated with coronary stenosis in patients with diabetes and metabolic syndrome. Vergeer *et al*. [26] has also previously identified a rare nonsynonymous mutation within *SCARB1* that reduced SR-BI function but did not reduce protein levels. These investigators did not observe an increased risk for atherosclerosis in family members carrying the risk allele for the missense mutation.

Although we and others have previously examined genetic associations with *SCARB1* SNPs, few studies have reported directly on the association of the common missense rs4238001 with cardiovascular risk factors and outcomes. One reason for the relative absence of reports on rs4238001 is that this particular SNP lies in a GC-rich region of the genome, making it challenging to design high-throughput genotyping chips typically used for large-scale genetic studies. For example, rs4238001 was selected for inclusion on the CARe IBC chip, but did not pass design. In the NHLBI Exome Sequencing Project [27], rs4238001 did not get adequate read depth to be selected for inclusion on the ExomeChip, even though it is a common variant with MAF > 0.05 in Whites, African Americans and Hispanics. For these reasons, few large-scale genetic consortia have reported results for this SNP. The public release of the 1,000 Genomes Phase 1 data has created the opportunity to impute such variants, and we are optimistic this resource will facilitate more wide-spread inclusion of rs4238001 in future genetic studies.

We previously reported that macrophages stably expressing rs4238001 showed enhanced degradation of SR-BI protein and significantly reduced selective cholesteryl-ester uptake from HDL [9]. The deleterious effect of this missense mutation on SR-BI protein levels and function has also been predicted based on bioinformatic approaches [27]. The Exome Variant Server (http://evs.gs.washington.edu/EVS/) produced by the NHLBI Exome Sequencing Project reports for rs4238001 a PolyPhen prediction of ‘probably damaging’ and a Genomic Evolutionary Rate Profiling (GERP) conservation score of 3.22, demonstrating conservation in this region of the protein. Bioinformatic analysis using HaploReg [28] revealed a number of nuclear proteins predicted to bind to rs4238001 including POL2, OCT2, POUI2F2, AP2ALPHA, AP2GAMMA, MXI1, RAD21, CCNT2, E2F6, and TAF1.

While we observed the rs4238001 T allele associated with increased LDL-C and LDL particle numbers in White males from MESA, the associations with CHD in an expanded set of n = 5,402 White males were based on regression models that accounted for LDL-C and other major cardiovascular risk factors. Therefore, our results suggest the rs4238001 SNP exerts independent effects on both LDL-C and CHD, with both effects playing a stronger role in males. These results suggest the effects of SR-BI on circulating lipoprotein cholesterol content, particle size and number in humans is not directly causal.

Acton *et al*. [8] previously reported that male carriers of the rs4238001 risk T allele residing in Zaragoza, Spain had significantly higher HDL-C levels. In contrast, we did not observe significant effects of the T allele on HDL-C levels in male participants of MESA. This might be attributable to our larger sample size (n = 201 men in the Spanish study) and/or to gene-dietary interactions between the two populations [29]. Investigators had previously shown that young adult carriers of the risk T allele had higher LDL-C levels when consuming a diet rich in saturated fats, suggesting an important role of SR-BI in human LDL-C metabolism [30]. It is also possible that alterations in cellular SR-BI protein expression and function might have more direct causal effects on atherosclerosis that have yet to be identified.

In summary, the common rs4238001 missense SNP in *SCARB1* was significantly associated with incident CHD in MESA participants, particularly men and African Americans. Analyses that incorporated independent population-based samples from three additional cohorts further underscored the importance of the association of rs4238001 on males, and White males in particular. Importantly, analyses within MESA that adjusted for traditional and nontraditional CVD risk factors did not attenuate this association, suggesting other pathways need to be explored to identify the causal one(s). The significant interaction of the rs4238001SNP with body mass index and the effects this interaction had on LpPLA2 mass and activity in African American women offers the possibility that inflammatory pathways might be key mediators in the causal pathway.

## Supporting Information

S1 Supporting Information Supplemental Methods. (DOC)

S1 Table Detailed results from meta-analysis across race/ethnic groups for survival analysis by Cox proportional hazards modeling CHD-Hard events on *SCARB1* SNP rs4238001 in MESA. (DOCX)

S2 Table Detailed race/ethnic-specific results for survival analysis by Cox proportional hazards modeling of CHD-Hard events on *SCARB1* SNP rs4238001 in MESA. (DOCX)

S3 Table Summary of lipid levels by rs4238001 genotype for MESA participants across three ethnic groups. (DOCX)

S4 Table Association of rs4238001 with inflammatory markers in MESA. (DOCX)

S5 Table Characteristics of participants across two ethnic groups by different cohorts. (DOCX)

S6 Table Power of association analyses for the full set of participants presented in Table 2. (DOCX)

S7 Table Genotype specific event counts within each cohort and race/ethnic group. (DOCX)
