# Supplementary material for: Association of the Lipoprotein Receptor SCARB1 Common Missense Variant rs4238001 with Incident Coronary Heart Disease
Source: PLoS One. 2015 May 20;10(5):e0125497. doi: 10.1371/journal.pone.0125497 (PMC4439156; doi:10.1371/journal.pone.0125497)
Supplement: S1 Supporting Information — (DOC) [file pone.0125497.s001.doc]

**Supporting Information for “Association of the lipoprotein receptor *SCARB1* common missense variant rs4238001 with incident coronary heart disease”**

Ani Manichaikul, Xin-Qun Wang, Solomon K. Musani, David M. Herrington, Wendy S. Post, James G. Wilson, Stephen S. Rich, Annabelle Rodriguez

**Phenotyping of MESA participants**

MESA participants had detailed medical histories (including medication and smoking history) and underwent examinations for anthropometry, blood pressure and vascular imaging (1). Fasting blood samples were taken for DNA, lipids and inflammatory biomarkers. Participants were asked to fast for 12h, avoid smoking in the morning of the examination, and avoid heavy exercise 12h before the examination. Measurements of mean HDL and LDL particle diameter and number were determined by nuclear magnetic resonance (NMR) spectroscopy by LipoScience (LipoScience Inc, North Carolina), as described previously (2). Both Lp-PLA_2_ mass and activity measurements were per­formed by diaDexus Inc. (South San Francisco, CA, USA), as described previously (3).

Blood samples collected at baseline and stored at –80°C until analysis were used for measurements of inflammatory markers. Interleukin-6 (IL-6), high-sensitivity C-reactive protein (hsCRP), and plasminogen activator inhibitor-1 (PAI-1) concentrations were measured at the Laboratory for Clinical Biochemistry Research (University of Vermont, Burlington, VT). The samples were processed with the use of a standardized protocol based on that used in the Cardiovascular Health Study (CHS) (26). Concentrations of plasma IL-6, E-selectin and soluble intercellular adhesion molecule-1 (sICAM-1) were measured using quantitative enzyme-linked immunosorbent assays (Quantikine HS Human IL-6 Immunoassay, Parameter Human sICAM-1 Immunoassay, Parameter Human sE-Selectin Immunoassay, respectively; R&D Systems, Minneapolis, MN) (27). Plasma homocysteine levels were measured using a fluorescence polarization immunoassay (IMx homocysteine assay, Axis Biochemicals ASA, Oslo, Norway) with the IMx analyzer (Abbott Diagnostics, Abbott Park, IL). PAI-1 levels were measured by ELISA (Diagnostica Stago, Inc., Parsippany, NJ).

In MESA, we defined CHD events to include incident MI, definite angina, probable angina (if followed by coronary artery bypass grafting or percutaneous coronary intervention), resuscitated cardiac arrest, or coronary heart disease death. For the purposes of this study, we defined CHD-All as probable or confirmed CHD events, CHD-Hard as confirmed CHD events, and incident MI (4).

**Genotype Data**

Participants recruited for the original MESA cohort (n=6,814) were genotyped in 2009 using the Affymetrix Human SNP array 6.0. Genotype quality control for these data has been described previously (5). Briefly, we filtered on SNP level call rate < 95%, individual level call rate < 95%, heterozygosity > 53%, and removed all monomorphic SNPs. The cleaned genotypic data were deposited with MESA phenotypic data into the database of Genotypes and Phenotypes (dbGaP) as the MESA SNP Health Association Resource (SHARe) project (study accession phs000209) for consenting individuals with 897,981 SNPs passing study specific quality control (QC).

**Principal component analysis**

We performed principal component analysis to adjust for population structure among MESA participants, as described previously (5). Briefly, we began by constructing subsets of typed SNPs, thinned for linkage disequilibrium (LD). Using the LD-thinned subsets of SNPs, we performed Principal Component Analysis (PCA) as implemented in the program SMARTPCA (6,7) to compute principal components (PCs) of ancestry for unrelated subsets of individuals, removing inferred first degree relatives from the analysis. We constructed histograms and QQ-plots to assess symmetry and normality of the distribution of loadings for each of the resulting PCs to determine the optimal number of PCs to include in genetic association analysis.

**Imputation quality of the *SCARB1* SNP rs4238001 in MESA**

Using the observed versus expected variance metric (8) to quantify imputation quality, we set a threshold at imputation quality > 0.5 for inclusion in the current investigation. The SNP rs4238001 was imputed with good quality in MESA Whites, African Americans, and Hispanics (imputation quality scores of 0.96, 0.85, and 0.87, respectively). We further set an inclusion threshold on minor allele frequency (MAF) > 0.05, and the SNP rs4238001 reached this threshold in Whites, African Americans and Hispanics (MAF of 0.110, 0.060, 0.098 respectively). Although the SNP was also imputed in MESA Chinese, the observed minor allele frequency was only 0.003. Therefore, MESA Chinese were not included in the current investigation.

**Genetic association analysis in MESA**

To select individuals for analysis, we began with the full MESA cohort, including participants from MESA Classic, MESA Family, and MESA Air. We stratified by race/ethnic group, and eliminated those individuals with top principal components of ancestry > 3.5 SD from the mean within any race/ethnic group. To allow study site to be included as a covariate in genetic association analysis within each race/ethnic group, we restricted the data set to individuals from study sites with data available for at least 20 individuals of that race/ethnic group. For each of the phenotypic analyses, we then restricted the data set to individuals with data available for the particular phenotype of interest.

We began with stratified analyses within each race/ethnic group. Within each race/ethnic group, we first constructed an unrelated subset of individuals by selecting at most one individual from each pedigree, and using a Cox proportional hazards model, as implemented in R (9). Genetic association analysis was conducted under an additive 1 df dosage model representing, for each individual, the expected number of copies of the risk allele T. In all analyses, we began with a basic model (**Model 1**) including age, sex, study site and principal components of ancestry. (Based on our examination of principal components within each race/ethnic group, as described above, we used 3 PCs for analysis of Whites, 1 PC for African Americans, and 3 PCs for Hispanics). To examine sensitivity of the results to risk factors of myocardial infarction (MI) and CHD, we further performed genetic association analysis under an extended model (**Model 2**) that added body mass index (BMI), diabetes status (yes/no, defined as combining treated diabetes and the 2003 American Diabetes Association (ADA) fasting criterion (10)), serum creatinine, LDL-C, HDL-C, hypertension status (yes/no, defined as according to the 1997 Joint National Committee (JNC) VI criterion (11)), education, smoking exposure (ever smoke [yes/no] and current smoke [yes/no]) to the basic regression model (**Model 1**). We checked the Cox proportional hazards assumption formally (12) and by visual inspection using the package R/survival to ensure there were no strong deviations (*P*>0.05) from this assumption for rs4238001 genotype dosage within each of the three ethnic groups.

In addition to Models 1 and 2, we examined results under other regression models to investigate the role of other potential confounders and/or mediators in the genetic association between rs4238001 and CHD. Additional regression models included: Model 2 + an indicator of lipid medication exposure at baseline (**Model 3**); and a model focused on lipid levels as measured by NMR spectroscopy, consisting of Model 2 + mean HDL particle diameter + HDL particle number + mean LDL particle diameter + LDL particle number (**Model 4)**.

**Validation cohorts**

We sought validation of the observed associations for the rs4238001 SNP through expanded analysis incorporating independent cohorts spanning both White and African American populations, including the Atherosclerosis Risk in Communities Study (ARIC), the Framingham Heart Study (FHS) and the Jackson Heart Study (JHS) for whom GWAS genotyping were available to allow imputation of the rs4238001 SNP.

*The Atherosclerosis Risk in Communities Study (ARIC)*: The ARIC study is a multi-center prospective investigation of atherosclerotic disease in a predominantly bi-racial population (13). White and African American men and women aged 45-64 years at baseline were recruited from 4 communities: Forsyth County, North Carolina; Jackson, Mississippi; suburban areas of Minneapolis, Minnesota; and Washington County, Maryland. A total of 15,792 individuals participated in the baseline examination in 1987-1989, with follow-up examinations in approximate 3-year intervals, during 1990-1992, 1993-1995, and 1996-1998. For the purpose of the current analysis, CHD events in ARIC were defined as MI, including silent MI detected by ECG, or CHD death.

Phenotype and genotype data were obtained from dbGaP for the GENEVA ARIC Project (study accession phs000090). From a total of 841,820 genotyped SNPs (Affy 6.0 array) on 13,113 participants, we applied quality control filters for SNP level call rate > 0.95, sample level call rate > 0.95, sample heterozygosity > 0.33, and sex mismatch. To complete imputation of *SCARB1* region SNPs, we identified 889 genotyped SNPs in the region of *SCARB1* (including flanking regions of about 1 Mb on either side, NCBI build 37 Chr 12 Pos 124.2 – 126.3 Mb), and used IMPUTE v2.3.0 (14) to complete imputation of additional SNPs in region from the 1,000 Genomes cosmopolitan Phase 1 v3 reference panel (15). Using the observed versus expected variance metric (8) to quantify imputation quality, we set a threshold at imputation quality > 0.5 for inclusion in the current investigation. The SNP rs4238001 was imputed with good quality in the ARIC Whites and African Americans (imputation quality scores of 0.97 and 0.92, respectively).

*The Framingham Heart Study (FHS):* The FHS was initiated in 1948 and comprised 5,209 men and women from the Framingham area who were between the ages of 28 and 62 years (16). Beginning in 1971, FHS enrolled 5,124 additional men and women, who were either offspring of the original cohort or spouses of those offspring. In 2002, a total of 4,095 third generation participants (men and women) were recruited. During each clinic exam cycle, the participants underwent a detailed examination including physical examination, medical history, laboratory testing, and electrocardiogram. Over the years, other tests (not necessarily performed at every exam cycle) have included pulmonary function, lifestyle, physical function, cognitive function questionnaires, and various noninvasive cardiovascular tests including echocardiograms.

To define CHD events with more complete information on covariates of interest, we used the Framingham Offspring Exam 6 as the “baseline” exam for the current investigation. We created a subset of participants comparable to MESA in terms of age and health status, we limited our investigation to Framingham Offspring participants age 45 or greater and free of CHD events (at Exam 6). For the purpose of the current analysis, CHD events were defined as MI recognized by echocardiogram (ECG) or without ECG but with enzymes and history, or autopsy evidence, unrecognized MI, angina pectoris, or CHD death.

Phenotype and genotype data were obtained from dbGaP for the Framingham SHARe Project (study accession phs000342). From a total of 500,568 genotyped SNPs (Affymetrix 500K) on 9,232 participants, we applied quality control filters for SNP level call rate > 0.95, sample level call rate > 0.95, sample heterozygosity > 0.32, and sex mismatch. To complete imputation of *SCARB1* region SNPs, we identified 477 genotyped SNPs in the region of *SCARB1* (including flanking regions of about 1 Mb on either side, NCBI build 37 Chr 12 Pos 124.2 – 126.3 Mb), and used IMPUTE v2.3.0 (14) to complete imputation of additional SNPs in region from the 1,000 Genomes cosmopolitan Phase 1 v3 reference panel (15). Using the observed versus expected variance metric (8) to quantify imputation quality, we set a threshold at imputation quality > 0.5 for inclusion in the current investigation. The SNP rs4238001 was imputed with good quality in the Framingham Offspring participants included in the current investigation (imputation quality score of 0.96).

*The Jackson Heart Study (JHS):* The Jackson Heart Study (JHS) is a prospective population-based study established in 2000 to study the causes of greater burden of cardiovascular diseases among African Americans. Four strategies were employed to recruit a total of 5,301 participants in the first examination cycle (2000 to 2004) from Jackson Mississippi metropolitan area counties of Hinds, Madison and Rankin (17); (i) all living Jackson MS participants enrolled in the Atherosclerosis Risk in Communities (ARIC) study aged 35-84 years at initiation of the study (31%) were invited to participate, also referred to as the ARIC-JHS overlap; (ii) a random sample (17%) recruited from a commercially available list (AccuData Integrated Marketing, Fort Myers, FL); (iii) a volunteer sample (30%) aged between 35-84 years who responded to targeted advertisements: radio, newspaper, local churches, and civic/social organizations; and (iv) family members (22%) of the first three groups who were at least 21 years old. Participants recruited in strategies ii-iv are referred to as the JHS-specific sample. Of the total recruited participants, 3,028 or 57.1 percent (892 ARIC-JHS overlap and 2,136 JHS-specific) gave consent that allowed their inclusion in genotyping through the CARe consortium (18) using the Affymetrix 6.0 Array (dbGaP study accession phs000499).

For this study, participants in the ARIC-JHS overlap group were excluded from the JHS analysis and were analyzed as ARIC participants. Genotyping quality control was performed as described (19), with exclusion of DNA samples with a genome-wide genotyping success rate <95%, SNPs with genotyping success rate <90%, monomorphic SNPs, and SNPs that map to several genomic locations. Additional filters were applied to remove SNPs with minor allele frequency (MAF) <1%, with genotyping success rate <95%, and SNPs where missingness can be predicted using surrounding haplotypes. Heterozygosity rates (in the form of inbreeding coefficients) on the autosomes were estimated to identify problematic DNA samples (poor DNA quality or contamination), and genome-wide genotype data were used to estimate identity-by-descent (IBD) between all pairwise combinations of samples to identify sample duplicates, contaminated samples, and cryptic relationships. Imputation was done using MaCH-Admix (20) with default parameter settings and the 1,000 Genomes cosmopolitan Phase 1 v3 reference panel (15). The imputation quality score for rs4238001 was 0.99.

**Expanded analysis across cohorts**

Genetic analyses were performed using Cox proportional hazards models under an additive model of inheritance. Regression models were constructed to follow as closely as possible with fully adjusted model (Model 2, described in “**Genetic association analysis in MESA**” above) used for main genetic analysis performed in MESA.

In FHS, detailed information on participant education was not available, so analyses were subset to include only those participants who answered “No” to the question “illiterate or low education”. Cox regression analysis in FHS proceeded as described above, with exclusion of participants with top PCs of ancestry > 3.5 SD from the mean. Covariates included age, sex, 3 PCs of ancestry, BMI, diabetes status (yes/no, defined as combining treated diabetes and the 2003 ADA fasting criterion (10)), serum creatinine, LDL-C as estimated by Friedwald’s equation (21), HDL-C, hypertension status (yes/no, defined as according to the 1997 JNC VI criterion (11)), smoking exposure (ever smoke [yes/no] and current smoke [yes/no]).

In ARIC and JHS, the covariates were defined mirroring the methods used for the fully adjusted model in MESA (Model 2, above), as each of these covariates could be retrieved according to the same definitions in these two cohorts.

Results from MESA were combined with those from the additional cohorts for expanded analysis within race/ethnic groups using fixed effect meta-analysis to combine estimated effects and standard errors across cohorts, as implemented in METAL (22).

**References**

1. Bild DE, Bluemke DA, Burke GL, Detrano R, Diez Roux AV, Folsom AR, Greenland P, Jacob DR, Jr., Kronmal R, Liu K, Nelson JC, O'Leary D, Saad MF, Shea S, Szklo M, Tracy RP. Multi-Ethnic Study of Atherosclerosis: Objectives and design. *Am J Epidemiol.* 2002;156:871-881.

2. Frazier-Wood AC, Manichaikul A, Aslibekyan S, Borecki IB, Goff DC, Hopkins PN, Lai CQ, Ordovas JM, Post WS, Rich SS, Sale MM, Siscovick D, Straka RJ, Tiwari HK, Tsai MY, Rotter JI, Arnett DK. Genetic variants associated with VLDL, LDL and HDL particle size differ with race/ethnicity. *Hum Genet*. 2013;132:405-13.

3. Garg PK, McClelland RL, Jenny NS, Criqui M, Liu K, Polak JF, Jorgensen NW, Cushman M. Association of lipoprotein-associated phospholipase A2 and endothelial function in the Multi-Ethnic Study of Atherosclerosis (MESA). *Vasc Med*. 2011;16:247-52.

4. Yeboah J, Folsom AR, Burke GL, Johnson C, Polak JF, Post W, Lima JA, Crouse JR, Herrington DM. Predictive value of brachial flow-mediated dilation for incident cardiovascular events in a population-based study: The multi-ethnic study of atherosclerosis. *Circulation*. 2009;120:502-509.

5. Manichaikul A, Naj AC, Herrington D, Post W, Rich SS, Rodriguez A. Association of SCARB1 variants with subclinical atherosclerosis and incident cardiovascular disease: The Multi-Ethnic Study of Atherosclerosis. *Arterioscler Thromb Vasc Biol.* 2012;32:1991-1999.

6. Patterson N, Price AL, Reich D. Population structure and eigenanalysis. *PLoS Genet.* 2006;2:e190.

7. Price AL, Patterson NJ, Plenge RM, Weinblatt ME, Shadick NA, Reich D. Principal components analysis corrects for stratification in genome-wide association studies. *Nat Genet.* 2006;38:904-909.

8. Li Y, Willer CJ, Ding J, Scheet P, Abecasis GR. MACH: Using sequence and genotype data to estimate haplotypes and unobserved genotypes. *Genet Epidemiol.* 2010;34:816-834.

9. R Development Core Team. R: A language and environment for statistical computing. R Foundation for Statistical Computing. Vienna, Austria, 2010. [http://www.R-project.org](http://www.R-project.org/).

10. Genuth S, Alberti KG, Bennett P, Buse J, Defronzo R, Kahn R, Kitzmiller J, Knowler WC, Lebovitz H, Lernmark A, Nathan D, Palmer J, Rizza R, Saudek C, Shaw J, Steffes M, Stern M, Tuomilehto J, Zimmet P. Follow-up report on the diagnosis of diabetes mellitus. *Diabetes Care*. 2003;26:3160-3167.

11. The sixth report of the joint national committee on prevention, detection, evaluation, and treatment of high blood pressure. *Arch Intern Med*. 1997;157:2413-2446.

12. Grambsch P, Therneau T. Proportional hazards tests and diagnostics based on weighted residuals. *Biometrika. 1994;*81:515-26.

13. The ARIC investigators. The Atherosclerosis Risk in Communities (ARIC) Study: design and objectives. *Am J Epidemiol*. 1989;129:687-702.

14. Howie BN, Donnelly P, Marchini J. A flexible and accurate genotype imputation method for the next generation of genome-wide association studies. *PLoS Genetics*. 2009;5: e1000529.

15. 1000 Genomes Project Consortium, Abecasis GR, Auton A, Brooks LD, DePristo MA, Durbin RM, Handsaker RE, Kang HM, Marth GT, McVean GA. An integrated map of genetic variation from 1,092 human genomes. *Nature*. 2012;491:56-65.

16. Dawber TR, Meadors GF, Moore FEJ. Epidemiological approaches to heart disease: the Framingham Study. *Am J Public Health.* 1951;41:279-286.

17. Taylor HA, Jr., Wilson JG, Jones DW, Sarpong DF, Srinivasan A, Garrison RJ, Nelson C, Wyatt SB. Toward resolution of cardiovascular health disparities in African Americans: design and methods of the Jackson Heart Study. *Ethn Dis*. 2005;15:S6-17.

18. Musunuru K, Lettre G, Young T, Farlow DN, Pirruccello JP, Ejebe KG, Keating BJ, Yang Q, Chen MH, Lapchyk N, Crenshaw A, Ziaugra L, Rachupka A, Benjamin EJ, Cupples LA, Fornage M, Fox ER, Heckbert SR, Hirschhorn JN, Newton-Cheh C, Nizzari MM, Paltoo DN, Papanicolaou GJ, Patel SR, Psaty BM, Rader DJ, Redline S, Rich SS, Rotter JI, Taylor HA, Jr., Tracy RP, Vasan RS, Wilson JG, Kathiresan S, Fabsitz RR, Boerwinkle E and Gabriel SB. Candidate gene association resource (CARe): design, methods, and proof of concept. *Circ Cardiovasc Genet*. 2010;3:267-275.

19. Lettre G, Palmer CD, Young T, Ejebe KG, Allayee H, Benjamin EJ, Bennett F, Bowden DW, Chakravarti A, Dreisbach A, Farlow DN, Folsom AR, Fornage M, Forrester T, Fox E, Haiman CA, Hartiala J, Harris TB, Hazen SL, Heckbert SR, Henderson BE, Hirschhorn JN, Keating BJ, Kritchevsky SB, Larkin E, Li M, Rudock ME, McKenzie CA, Meigs JB, Meng YA, Mosley TH, Newman AB, Newton-Cheh CH, Paltoo DN, Papanicolaou GJ, Patterson N, Post WS, Psaty BM, Qasim AN, Qu L, Rader DJ, Redline S, Reilly MP, Reiner AP, Rich SS, Rotter JI, Liu Y, Shrader P, Siscovick DS, Tang WH, Taylor HA, Tracy RP, Vasan RS, Waters KM, Wilks R, Wilson JG, Fabsitz RR, Gabriel SB, Kathiresan S, Boerwinkle E. Genome-Wide Association Study of Coronary Heart Disease and Its Risk Factors in 8,090 African Americans: The NHLBI CARe Project. *PLoS Genet*. 2011;7:e1001300.

20. Liu EY, Li M, Wang W, Li Y. MaCH-Admix: Genotype Imputation for Admixed Populations. *Genet Epidemiol*. 2013;37:25-37.

21. Friedewald WT, Levy RI, Fredrickson DS. Estimation of the concentration of low-density lipoprotein cholesterol in plasma, without use of the preparative ultracentrifuge. *Clin Chem*. 1972; 18:499–502.

22. Willer CJ, Li Y, Abecasis GR. METAL: Fast and efficient meta-analysis of genomewide association scans. *Bioinformatics.* 2010;26:2190-2191.
