# Supplementary material for: Association of the Lipoprotein Receptor SCARB1 Common Missense Variant rs4238001 with Incident Coronary Heart Disease
Source: PLoS One. 2015 May 20;10(5):e0125497. doi: 10.1371/journal.pone.0125497 (PMC4439156; doi:10.1371/journal.pone.0125497)
Supplement: S1 Table — (DOCX) [file pone.0125497.s002.docx]

**S1 Table. Supplemental Table 1:** Detailed results from meta-analysis across race/ethnic groups for survival analysis by Cox proportional hazards modeling CHD-Hard events on *SCARB1* SNP rs4238001 in MESA.

|  | **All** | | | **Male** | | | **Female** | | |
| --- | --- | --- | --- | --- | --- | --- | --- | --- | --- |
| **Model** | Beta | SE | P-value | Beta | SE | P-value | Beta | SE | P-value |
| Model 1 (basic) | 0.383 | 0.181 | 0.034 | 0.458 | 0.215 | 0.033 | 0.278 | 0.340 | 0.413 |
| Model 2 (extended) | 0.402 | 0.183 | 0.028 | 0.562 | 0.222 | 0.011 | 0.298 | 0.373 | 0.424 |
| Model 3 (Model 2 + lipid meds.) | 0.393 | 0.184 | 0.032 | 0.558 | 0.223 | 0.012 | 0.252 | 0.379 | 0.506 |
| Model 4 (Model 2 + NMR lipids) | 0.412 | 0.184 | 0.023 | 0.579 | 0.224 | 0.010 | 0.313 | 0.389 | 0.421 |

Estimated effects are reported for rs4238001 effect allele T (versus the reference allele C) under the following models of adjustment: Model 1 (basic), Model 2 (extended), Model 3 (Model 2 + lipid medication), and Model 4 (Model 2 + NMR lipids). Analyses were conducted stratified by race/ethnic group and combined by meta-analysis, for all participants as well as stratified by sex (males or female).
