# Supplementary material for: Association of the Lipoprotein Receptor SCARB1 Common Missense Variant rs4238001 with Incident Coronary Heart Disease
Source: PLoS One. 2015 May 20;10(5):e0125497. doi: 10.1371/journal.pone.0125497 (PMC4439156; doi:10.1371/journal.pone.0125497)
Supplement: S2 Table — (DOCX) [file pone.0125497.s003.docx]

**S2 Table. Supplemental Table 2:** Detailed race/ethnic-specific results for survival analysis by Cox proportional hazards modeling of CHD-Hard events on *SCARB1* SNP rs4238001 in MESA.

|  |  | **All** | | | | **Male** | | | | **Female** | | | |
| --- | --- | --- | --- | --- | --- | --- | --- | --- | --- | --- | --- | --- | --- |
| **Model** | **Group** | N  (events) | Beta | SE | P-value | N  (events) | Beta | SE | P-value | N  (events) | Beta | SE | P-value |
| **Model 1 (basic)** | White | 2319  (77) | 0.201 | 0.252 | 0.424 | 1111  (52) | 0.442 | 0.282 | 0.117 | 1208  (25) | -0.413 | 0.560 | 0.461 |
|  | African American | 1570  (46) | 0.721 | 0.350 | 0.039 | 729  (30) | 0.467 | 0.496 | 0.346 | 841  (16) | 0.957 | 0.501 | 0.056 |
|  | Hispanic | 1292  (40) | 0.397 | 0.386 | 0.304 | 637  (29) | 0.493 | 0.444 | 0.267 | 655  (11) | -0.058 | 0.817 | 0.943 |
| **Model 2 (extended)** | White | 2275  (77) | 0.194 | 0.253 | 0.442 | 1088  (52) | 0.556 | 0.293 | 0.058 | 1187  (25) | -0.432 | 0.571 | 0.449 |
|  | African American | 1533  (46) | 0.766 | 0.359 | 0.033 | 715  (30) | 0.539 | 0.503 | 0.284 | 818  (16) | 1.279 | 0.574 | 0.027 |
|  | Hispanic | 1255  (39) | 0.469 | 0.395 | 0.235 | 618  (29) | 0.593 | 0.463 | 0.200 | 637  (10) | -0.367 | 0.967 | 0.704 |
| **Model 3 (Model 2 + lipid meds.)** | White | 2275  (77) | 0.192 | 0.253 | 0.449 | 1088  (52) | 0.548 | 0.293 | 0.061 | 1187  (25) | -0.441 | 0.573 | 0.442 |
|  | African American | 1531  (46) | 0.761 | 0.361 | 0.035 | 715  (30) | 0.531 | 0.505 | 0.294 | 816  (16) | 1.269 | 0.573 | 0.027 |
|  | Hispanic | 1255  (39) | 0.446 | 0.397 | 0.262 | 618  (29) | 0.607 | 0.464 | 0.191 | 637  (10) | -0.892 | 1.074 | 0.407 |
| **Model 4 (Model 2 + NMR lipids)** | White | 2267  (76) | 0.199 | 0.254 | 0.433 | 1087  (52) | 0.538 | 0.294 | 0.068 | 1180  (24) | -0.433 | 0.569 | 0.447 |
|  | African American | 1526  (46) | 0.834 | 0.363 | 0.022 | 710  (30) | 0.531 | 0.514 | 0.301 | 816  (16) | 1.394 | 0.599 | 0.020 |
|  | Hispanic | 1249  (39) | 0.453 | 0.396 | 0.253 | 616  (29) | 0.638 | 0.465 | 0.170 | 633  (10) | -0.626 | 1.149 | 0.586 |

Estimated effects are reported for rs4238001 effect allele T (versus the reference allele C) under the following models of adjustment: Model 1 (basic), Model 2 (extended), Model 3 (Model 2 + lipid medication), and Model 4 (Model 2 + NMR lipids). Analyses were conducted stratified by race/ethnic group for all participants as well as stratified by sex (males or female).
