# Supplementary material for: Association of the Lipoprotein Receptor SCARB1 Common Missense Variant rs4238001 with Incident Coronary Heart Disease
Source: PLoS One. 2015 May 20;10(5):e0125497. doi: 10.1371/journal.pone.0125497 (PMC4439156; doi:10.1371/journal.pone.0125497)
Supplement: S3 Table — (DOCX) [file pone.0125497.s004.docx]

**S3 Table. Supplemental Table 3:** Summary of lipid levels by rs4238001 genotype for MESA participants across three ethnic groups.

|  | Caucasian | African American | Hispanic |
| --- | --- | --- | --- |
| rs4238001 homozygotes (C/C)* |  |  |  |
| No. subjects | 1852 | 1397 | 1062 |
| Women | 953 (51.5) | 751 (53.8) | 543 (51.1) |
| Triglycerides, mg/dL | 114 [77, 165] | 90 [66, 123] | 135 [95, 190] |
| HDL-C, mg/dL | 50 [41, 61] | 50 [42, 61] | 45 [39, 54] |
| LDL-C, mg/dL | 114 [94, 135] | 115 [95, 136] | 118 [97, 139] |
|  |  |  |  |
| rs4238001 T allele carriers* |  |  |  |
| No. subjects | 467 | 173 | 230 |
| Women | 255 (54.6) | 90 (52.0) | 112 (48.7) |
| Triglycerides, mg/dL | 114 [76, 159] | 94 [67, 123] | 142 [98, 200] |
| HDL-C, mg/dL | 50 [42, 62] | 48 [41, 60] | 46 [39, 53] |
| LDL-C, mg/dL | 120 [101, 139] | 113 [88, 134] | 120 [102, 140] |

Data are presented as N (%) for binary measures or median [IQR] for continuous measure.

*Genotypes for rs4238001 were obtained by imputation, and genotype dosage (estimated number of copies of the T allele) was used as a proxy to classify participants as homozygotes (C/C, dosage < 0.5) vs. T allele carriers (dosage ≥ 0.5).
