# Supplementary material for: Association of the Lipoprotein Receptor SCARB1 Common Missense Variant rs4238001 with Incident Coronary Heart Disease
Source: PLoS One. 2015 May 20;10(5):e0125497. doi: 10.1371/journal.pone.0125497 (PMC4439156; doi:10.1371/journal.pone.0125497)
Supplement: S4 Table — (DOCX) [file pone.0125497.s005.docx]

**S4 Table. Supplemental Table 4:** Association of rs4238001 with inflammatory markers in MESA.

|  |  | **All** | | | | **Male** | | | | **Female** | | | |
| --- | --- | --- | --- | --- | --- | --- | --- | --- | --- | --- | --- | --- | --- |
| **Trait (units)** | **Group** | N | Beta | SE | P-value | N | Beta | SE | P-value | N | Beta | SE | P-value |
| **IL-6***  **(log pg/mL)** | White | 2277 | -0.027 | 0.031 | 0.383 | 1093 | -0.064 | 0.044 | 0.148 | 1184 | 0.011 | 0.044 | 0.800 |
|  | African American | 1503 | 0.038 | 0.053 | 0.470 | 701 | 0.065 | 0.080 | 0.418 | 802 | 0.030 | 0.070 | 0.675 |
|  | Hispanic | 1259 | -0.068 | 0.044 | 0.122 | 618 | -0.124 | 0.063 | 0.051 | 641 | -0.006 | 0.060 | 0.916 |
|  | Meta-analysis |  | -0.026 | 0.023 | 0.254 |  | -0.058 | 0.033 | 0.077 |  | 0.010 | 0.032 | 0.752 |
| **E-selectin**  **(ng/mL)** | White | 553 | 0.891 | 1.818 | 0.624 | 259 | 2.540 | 2.757 | 0.358 | 294 | -0.703 | 2.461 | 0.775 |
|  | African American | 223 | -3.044 | 6.781 | 0.654 | 78 | -1.128 | 9.265 | 0.903 | 145 | -1.885 | 10.55 | 0.858 |
|  | Hispanic | 262 | 4.080 | 4.316 | 0.345 | 119 | 2.948 | 5.353 | 0.583 | 143 | 5.788 | 6.998 | 0.410 |
|  | Meta-analysis |  | 1.118 | 1.627 | 0.492 |  | 2.380 | 2.370 | 0.315 |  | -0.076 | 2.268 | 0.973 |
| **sICAM-1**  **(ng/mL)** | White | 1249 | -0.101 | 3.870 | 0.979 | 574 | -0.510 | 5.513 | 0.926 | 675 | 1.727 | 5.345 | 0.747 |
|  | African American | 451 | 19.13 | 16.85 | 0.257 | 179 | 43.27 | 25.76 | 0.095 | 272 | 6.540 | 23.05 | 0.777 |
|  | Hispanic | 557 | 10.32 | 8.09 | 0.203 | 241 | 0.91 | 12.05 | 0.940 | 316 | 18.21 | 10.89 | 0.096 |
|  | Meta-analysis |  | 2.550 | 3.418 | 0.456 |  | 1.324 | 4.920 | 0.788 |  | 4.99 | 4.70 | 0.288 |
| **PAI-1***  **(log ng/mL)** | White | 400 | 0.118 | 0.108 | 0.273 | 184 | 0.098 | 0.162 | 0.545 | 216 | 0.113 | 0.147 | 0.444 |
|  | African American | 180 | -0.101 | 0.250 | 0.686 | 63 | -0.057 | 0.351 | 0.871 | 117 | 0.072 | 0.375 | 0.848 |
|  | Hispanic | 209 | 0.301 | 0.157 | 0.057 | 98 | 0.302 | 0.197 | 0.129 | 111 | 0.243 | 0.263 | 0.359 |
|  | Meta-analysis |  | 0.146 | 0.084 | 0.082 |  | 0.153 | 0.118 | 0.193 |  | 0.136 | 0.122 | 0.263 |
| **hsCRP***  **(log mg/L)** | White | 2452 | -0.019 | 0.052 | 0.712 | 1181 | -0.012 | 0.071 | 0.867 | 1271 | -0.014 | 0.075 | 0.854 |
|  | African American | 1949 | -0.127 | 0.084 | 0.129 | 882 | -0.101 | 0.123 | 0.409 | 1067 | -0.132 | 0.113 | 0.243 |
|  | Hispanic | 1739 | 0.006 | 0.065 | 0.921 | 826 | -0.100 | 0.092 | 0.275 | 913 | 0.080 | 0.089 | 0.371 |
|  | Meta-analysis |  | -0.032 | 0.037 | 0.386 |  | -0.055 | 0.051 | 0.284 |  | -0.007 | 0.051 | 0.887 |
| **Homocysteine***  **(log mmol/L)** | White | 2305 | 0.008 | 0.012 | 0.505 | 1109 | 0.006 | 0.018 | 0.723 | 1196 | 0.007 | 0.018 | 0.709 |
|  | African American | 1556 | 0.007 | 0.022 | 0.754 | 719 | 0.008 | 0.033 | 0.807 | 837 | 0.002 | 0.029 | 0.938 |
|  | Hispanic | 1287 | 0.016 | 0.018 | 0.379 | 634 | 0.039 | 0.025 | 0.126 | 653 | -0.015 | 0.026 | 0.551 |
|  | Meta-analysis |  | 0.010 | 0.009 | 0.279 |  | 0.016 | 0.013 | 0.243 |  | 0.000 | 0.013 | 0.994 |

Estimated effects are reported for rs4238001 effect allele T (versus the reference allele C) under a basic model of association (Model 1).

*IL-6, PAI-1, hsCRP and homocysteine levels were analyzed under a log-transform.
