# Supplementary material for: Association of the Lipoprotein Receptor SCARB1 Common Missense Variant rs4238001 with Incident Coronary Heart Disease
Source: PLoS One. 2015 May 20;10(5):e0125497. doi: 10.1371/journal.pone.0125497 (PMC4439156; doi:10.1371/journal.pone.0125497)
Supplement: S5 Table — (DOCX) [file pone.0125497.s006.docx]

**S5 Table. Supplemental Table 5:** Characteristics of participants across two ethnic groups by different cohorts.

|  | **White** | | **African American** | |
| --- | --- | --- | --- | --- |
| **Participant characteristics*** | **ARIC Whites** | **FHS** | **ARIC African**  **American** | **JHS** |
| No. subjects | 7465 | 2217 | 2299 | 2138 |
| Women | 4128 (55%) | 1240 (56%) | 1442 (63%) | 1290 (60.6) |
| Age, years | 54  [49, 59] | 58  [52, 66] | 53  [48, 58] | 49  [42, 56] |
| BMI, kg/m^2^ | 26.1  [23.5, 29.4] | 27.3  [24.5, 30.7] | 28.8  [25.5, 32.8] | 30.8  [27.1, 36.1] |
|  |  |  |  |  |
| Education: completed high school | 6364 (85.3) | N/A | 1389 (60.4) | 1903 (89.5) |
| Education: completed technical degree, associate degree, bachelor's degree or higher | 2905 (38.9) | N/A | 744 (32.4) | 902 (42.4) |
|  |  |  |  |  |
| HDL-C, mg/dL | 48.2 [39.5, 60.7] | 50.0 [40.0, 61.0] | 53.0 [43.0, 64.5] | 48  [40, 57] |
| LDL-C, mg/dL | 134.9 [111.6, 159.8] | 125.6 [104.8, 148.6] | 134.9 [108.7, 162.2] | 124  [100, 147] |
| Serum creatinine, mg/dL | 1.10 [1.00, 1.20] | 1.04 [0.90, 1.18] | 1.10 [1.00, 1.20] | 0.94  [0.85, 1.13] |
|  |  |  |  |  |
| Diabetes (yes/no) | 561 (8) | 193 (9) | 414 (18) | 336 (16.0) |
| Hypertension (yes/no) | 910 (12) | 526 (24) | 688 (30) | 1203 (56.9) |
|  |  |  |  |  |
| Ever smoke (yes/no) | 4395 (59) | 1326 (60) | 1228 (53) | 661 (31.2) |
| Current smoke (yes/no) | 1843 (25) | 309 (14) | 676 (29) | 311 (14.6) |
|  |  |  |  |  |
| Lipid medication (yes/no) | 209 (2.8) | 237 (10.7) | 25 (1.1) | 187 (9.5) |
|  |  |  |  |  |
| **Clinical events** |  |  |  |  |
| CHD-Hard (define as MI + CHD death) | 618 (8.3) | 176 (8.0) | 246 (10.7) | 63 (3.0) |
| Myocardial infarction | 519 (7.0) | 95 (4.3) | 169 (7.4) | 55 (2.6) |
| Follow-up time (years) | 16.1  [15.3, 16.9] | 9.1  [8.2, 10.0] | 15.8  [15.0, 16.8] | 7.9  [7.2, 8.6] |

Data are presented as N (%) for binary measures or median [IQR] for continuous measure.

*Summary statistics are reported for the subset of individuals with data available for at least one of the clinical events.
