# Supplementary material for: Association of the Lipoprotein Receptor SCARB1 Common Missense Variant rs4238001 with Incident Coronary Heart Disease
Source: PLoS One. 2015 May 20;10(5):e0125497. doi: 10.1371/journal.pone.0125497 (PMC4439156; doi:10.1371/journal.pone.0125497)
Supplement: S6 Table — (DOCX) [file pone.0125497.s007.docx]

**S6 Table. Supplemental Table 6:** Power of association analyses for the full set of participants presented in Table 2.

|  |  | **rs4238001** | Combined  (MESA + Additional cohorts) | | | |
| --- | --- | --- | --- | --- | --- | --- |
|  | **Group** | **MAF**† | N  (events) | Beta=0.05  (OR=1.05) | Beta=0.10  (OR=1.11) | Beta=0.20  (OR=1.22) |
| All | White | 0.104 | 11957  (871) | 9.3% | 26.0% | 72.2% |
|  | African American | 0.055 | 5962  (355) | 6.0% | 9.7% | 23.4% |
|  | Hispanic | 0.098 | 1255  (39) | 5.2% | 5.9% | 8.4% |
|  | Meta-analysis |  | 19174  (1255) | 10.4% | 31.2% | 81.5% |
| Male | White | 0.103 | 5402  (554) | 7.6% | 17.6% | 51.0% |
|  | African American | 0.053 | 2417  (180) | 5.5% | 7.2% | 13.7% |
|  | Hispanic | 0.103 | 618  (29) | 5.1% | 5.7% | 7.6% |
|  | Meta-analysis |  | 8432  (758) | 8.1% | 20.3% | 58.9% |
| Female | White | 0.105 | 6555  (317) | 6.6% | 12.7% | 34.8% |
|  | African American | 0.056 | 3546  (175) | 5.5% | 7.4% | 14.2% |
|  | Hispanic | 0.094 | 637  (10) | 5.1% | 5.2% | 5.9% |
|  | Meta-analysis |  | 10742  (507) | 7.2% | 15.6% | 45.2% |

†MAF denotes Minor Allele Frequency

Power calculations were completed in QUANTO (1) based on logistic regression analysis for the stated effects (Beta) and corresponding odds ratios (ORs), minor alleles frequencies (MAF), sample sizes, numbers of events and assuming a nominal Type I error rate of α=0.05. The ORs included in power analysis reflect a realistic range of SNP effects presented in recent genome-wide studies of CHD (2). Power calculation for meta-analysis across race/ethnic groups assumes the weighted mean MAF of 0.088.

**References**

1. Gauderman WJ and Morrison JM. QUANTO 1.1: A computer program for power and sample size calculations for genetic-epidemiology studies. [http://hydra.usc.edu/gxe]. 2006.

2. Deloukas P, Kanoni S, Willenborg C, Farrall M, Assimes TL, Thompson JR, Ingelsson E, Saleheen D, Erdmann J, Goldstein BA, Stirrups K, Konig IR, Cazier JB, Johansson A, Hall AS, Lee JY, Willer CJ, Chambers JC, Esko T, Folkersen L, Goel A, Grundberg E, Havulinna AS, Ho WK, Hopewell JC, Eriksson N, Kleber ME, Kristiansson K, Lundmark P, Lyytikainen LP, Rafelt S, Shungin D, Strawbridge RJ, Thorleifsson G, Tikkanen E, Van Zuydam N, Voight BF, Waite LL, Zhang W, Ziegler A, Absher D, Altshuler D, Balmforth AJ, Barroso I, Braund PS, Burgdorf C, Claudi-Boehm S, Cox D, Dimitriou M, Do R, Doney AS, El Mokhtari N, Eriksson P, Fischer K, Fontanillas P, Franco-Cereceda A, Gigante B, Groop L, Gustafsson S, Hager J, Hallmans G, Han BG, Hunt SE, Kang HM, Illig T, Kessler T, Knowles JW, Kolovou G, Kuusisto J, Langenberg C, Langford C, Leander K, Lokki ML, Lundmark A, McCarthy MI, Meisinger C, Melander O, Mihailov E, Maouche S, Morris AD, Muller-Nurasyid M, Nikus K, Peden JF, Rayner NW, Rasheed A, Rosinger S, Rubin D, Rumpf MP, Schafer A, Sivananthan M, Song C, Stewart AF, Tan ST, Thorgeirsson G, van der Schoot CE, Wagner PJ, Wells GA, Wild PS, Yang TP, Amouyel P, Arveiler D, Basart H, Boehnke M, Boerwinkle E, Brambilla P, Cambien F, Cupples AL, de Faire U, Dehghan A, Diemert P, Epstein SE, Evans A, Ferrario MM, Ferrieres J, Gauguier D, Go AS, Goodall AH, Gudnason V, Hazen SL, Holm H, Iribarren C, Jang Y, Kahonen M, Kee F, Kim HS, Klopp N, Koenig W, Kratzer W, Kuulasmaa K, Laakso M, Laaksonen R, Lind L, Ouwehand WH, Parish S, Park JE, Pedersen NL, Peters A, Quertermous T, Rader DJ, Salomaa V, Schadt E, Shah SH, Sinisalo J, Stark K, Stefansson K, Tregouet DA, Virtamo J, Wallentin L, Wareham N, Zimmermann ME, Nieminen MS, Hengstenberg C, Sandhu MS, Pastinen T, Syvanen AC, Hovingh GK, Dedoussis G, Franks PW, Lehtimaki T, Metspalu A, Zalloua PA, Siegbahn A, Schreiber S, Ripatti S, Blankenberg SS, Perola M, Clarke R, Boehm BO, O'Donnell C, Reilly MP, Marz W, Collins R, Kathiresan S, Hamsten A, Kooner JS, Thorsteinsdottir U, Danesh J, Palmer CN, Roberts R, Watkins H, Schunkert H and Samani NJ. Large-scale association analysis identifies new risk loci for coronary artery disease. *Nat Genet.* 2013;45:25-33.
