# Supplementary material for: Association of the Lipoprotein Receptor SCARB1 Common Missense Variant rs4238001 with Incident Coronary Heart Disease
Source: PLoS One. 2015 May 20;10(5):e0125497. doi: 10.1371/journal.pone.0125497 (PMC4439156; doi:10.1371/journal.pone.0125497)
Supplement: S7 Table — (DOCX) [file pone.0125497.s008.docx]

**S7 Table. Supplemental Table 7:** Genotype specific event counts within each cohort and race/ethnic group.

|  |  | **Group** | | | | | |
| --- | --- | --- | --- | --- | --- | --- | --- |
|  |  | **White** | | **African American** | | **Hispanic** | |
|  | **rs4238001 genotype*** | **CC** | **T+** | **CC** | **T+** | **CC** | **T+** |
| **Cohort** | **MESA** | 61 | 16 | 37 | 9 | 31 | 8 |
|  | **ARIC** | 505 | 113 | 220 | 26 | - | - |
|  | **FHS** | 123 | 53 | - | - | - | - |
|  | **JHS** | - | - | 58 | 5 | - | - |
|  | **Total** | 689 | 182 | 315 | 40 | 31 | 8 |

*Genotypes for rs4238001 are classified according to genotype dosage for the T allele <0.5 (homozygotes CC) vs. ≥ 0.5 (carriers of the minor allele, T+).
